# Awareness of lung cancer among urban residents in Sichuan Province and its impact on their willingness to choose medical institutions for cancer screening

**DOI:** 10.3389/fpubh.2024.1388140

**Published:** 2025-01-07

**Authors:** Qi Chai, Ruicen Li, Ting Bao, Zhibo Yang, Qing Liu, Feng Chen

**Affiliations:** ^1^Integrated Care Management Center, West China Hospital, Sichuan University, Chengdu, China; ^2^Department of Health Management Center, West China Hospital, Sichuan University, Chengdu, China

**Keywords:** awareness of lung cancer, cancer screening, county or below medical institutions, hierarchical diagnosis and treatment system, propensity score matching

## Abstract

**Introduction:**

This study aimed to investigate the current level of knowledge about lung cancer among urban residents in Sichuan Province and to assess its influence on their willingness to choose county-level or lower-level medical institutions for cancer screening.

**Methods:**

A total of 31,184 urban residents of Sichuan Province were included in the cross-sectional study. Binary logistic regression and propensity score matching (PSM) were used to assess the influence effect.

**Results:**

The results showed that (1) only 23.88% of the residents self-reported having good knowledge about lung cancer. They mainly acquired knowledge from the media (43%) and medical staff (42%). Only 33.5% of the participants had undergone lung cancer screening, with the main reasons being periodic physical examinations (54%) and physician recommendations (23%). (2) Binary logistic regression analysis revealed that knowledge of lung cancer was significantly associated with the participants’ willingness to undergo lung cancer screening at county-level or lower-level medical institutions [OR = 1.185, 95% CI (1.113 -1.263), *p* < 0.001]. (3) Using PSM, it was found that the willingness of residents who had good knowledge of lung cancer-related topics increased by 2.8% after using kernel matching, by 3.1% after using one-to-one nearest neighbor matching, and by 2.4% after using radius matching with a caliper size of 0.001. (4) After stratifying by psychological status, we found that among residents with unstable psychological status, the willingness of those who had good knowledge of lung cancer increased by 5.3% after using kernel matching, by 3.6% after using one-to-one nearest neighbor matching, and by 4.9% after using radius matching with a caliper size of 0.001.

**Discussion:**

Improving urban residents’ understanding of the disease could help improve the current situation of hierarchical diagnosis and treatment.

**Systematic review registration:**

https://www.crd.york.ac.uk/prospero/, identifier CRD42024556625.

## 1 Introduction

Cancer is one of the leading causes of death and economic burden in China (1). The combination of early detection, diagnosis, and treatment is key to achieving higher efficiency in China’s healthcare system, particularly in the context of limited resources (2). Currently, the majority of cancer screening techniques are simple, and common cancer screening modalities are available at all levels of medical institutions. For instance, the ThinPrep cytology test for cervical cancer screening and the fecal occult blood test for colorectal cancer screening are commonly available in county-level or lower-level medical institutions (3, 4). Encouraging patients to seek medical services at these institutions could play a significant role in regulating patients’ therapeutic plans, controlling medical and healthcare expenditures, and improving the efficiency of medical and healthcare services (5–7). Moreover, enhancing the capacity of county-level or lower-level medical institutions for cancer screening can reduce the burden on larger hospitals and ensure coverage for the majority of residents.

Lung cancer is the leading cause of cancer-related morbidity and mortality in China (8, 9). It has the highest incidence rate in China, placing a significant social and economic burden on the public health system. Numerous studies have shown that low-dose spiral CT (LDCT) is associated with significantly lower lung cancer mortality and all-cause mortality rates in China (10–12). However, due to China’s large population, implementing lung cancer screening programs requires substantial efforts. Lung cancer screening in county-level or lower-level medical institutions can significantly reduce the burden on high-level hospitals by targeting high-risk groups. This approach also improves the allocation of medical resources.

In October 2014, Sichuan Province officially implemented the hierarchical medical system, aiming to address the structural imbalance in the allocation of medical and healthcare resources. However, some studies have indicated that the proportion of primary care visits has not significantly increased since the implementation of the policy (13). Upon reviewing the recent literature on the hierarchical medical system, we found that few studies have examined the impact of residents’ disease awareness on their choice of medical institutions. Numerous behavioral theories suggest that cognition is closely related to behavior (14–17). Based on this, we proposed a hypothesis that residents’ awareness of the disease may influence their choice of clinical medical institutions. According to the Appraisal-Tendency Framework (ATF), anxious individuals exhibit distinct behavioral preferences in decision-making, often opting for more conservative and safer strategies to reduce uncertainty and minimize potential negative outcomes (18, 19). Therefore, we hypothesized that residents with different mental states may make different medical treatment decisions.

To test our hypothesis, this study aimed to explore how urban residents’ awareness of lung cancer influences their choice of medical institution for screening. In addition, it assessed the participants’ willingness based on their psychological stability.

## 2 Materials and methods

### 2.1 Participants

This study was cross-sectional, and convenience sampling was employed, with each participant completing a questionnaire. From March to November 2021, a simple random sampling method was used to select residents from physical examination centers in hospitals across various municipalities in Sichuan Province. Sichuan Province has 21 municipalities; however, due to the limited population in Ganzi Prefecture and Aba Prefecture, participants were randomly selected from the remaining 19 municipalities based on the total resident count. The resident population data were obtained from the *Sichuan Provincial Health Statistical Yearbook 2020* (20). The paper version of the questionnaire was sent to the residents by the investigators. After removing some missing values, 31,184 participants remained. Criteria were as follows: (1) age greater than 18 years; (2) voluntary participation; and (3) availability of CT equipment and professional imaging physicians for lung cancer screening at county-level or lower-level medical institutions, where the residents live.

### 2.2 Data collection

This study used a self-assessment questionnaire to collect data on the following: age, sex, ethnicity, education level, occupation, type of medical insurance, commercial insurance status, average income per person per month, acceptable screening costs, smoking status, family history of cancer, knowledge about lung cancer, willingness to undergo lung cancer screening at county-level or lower-level medical institutions, and psychological status. In this study, the acceptable screening costs for lung cancer screening were measured by the question: “How much is the most you are willing to pay for lung cancer screening?” The responses were categorized into the following five levels: less than 100 yuan, 101–200 yuan, 201–300 yuan, 301–400 yuan, and more than 400 yuan. Their knowledge about lung cancer was measured by the following yes–no question: “Do you know about lung cancer?” Their willingness to undergo lung cancer screening at a county-level medical community institution was measured by the following yes–no question: “Are you willing to choose county-level or lower-level medical institutions for lung cancer screening?” The psychological status was measured by the following yes–no question: “Do you feel anxious because of abnormal physical examination results?”

### 2.3 Statistical analysis

SPSS version 23 was used for data analysis. Data with a normal distribution were presented as mean ± SD. Student’s *t*-test was used to compare two independent samples. Qualitative features were presented as frequencies and percentages and compared using the chi-squared test. Binary logistic regression was used for multi-factor analysis.

After the binary logistic regression analysis, propensity score matching (PSM) was performed for robustness testing using Stata 15.0. The propensity score (PS) for an individual is defined as the probability of being assigned to the “treatment” group, given all relevant covariates. The PS is typically estimated using a logistic regression model that incorporates all variables that may be related to the outcome and/or the treatment decision (21, 22). In this study, knowledge of lung cancer was treated as an independent variable. We grouped the residents according to this variable. The outcome was a willingness to undergo lung cancer screening at county-level or lower-level medical institutions. The covariate factors included age, sex, ethnicity, education level, occupation, type of medical insurance, commercial insurance, average income per person per month, acceptable screening cost for lung cancer screening, smoking status, and family history of cancer.

We used kernel matching as the main method. To verify whether different matching methods affected the results, 1:1 nearest neighbor matching and radius matching with a caliper size of 0.001 were applied. Finally, psychological status was used as a stratification variable, and the participants were grouped based on their psychological status.

## 3 Results

### 3.1 Awareness of lung cancer-related knowledge

**Figure 1:**
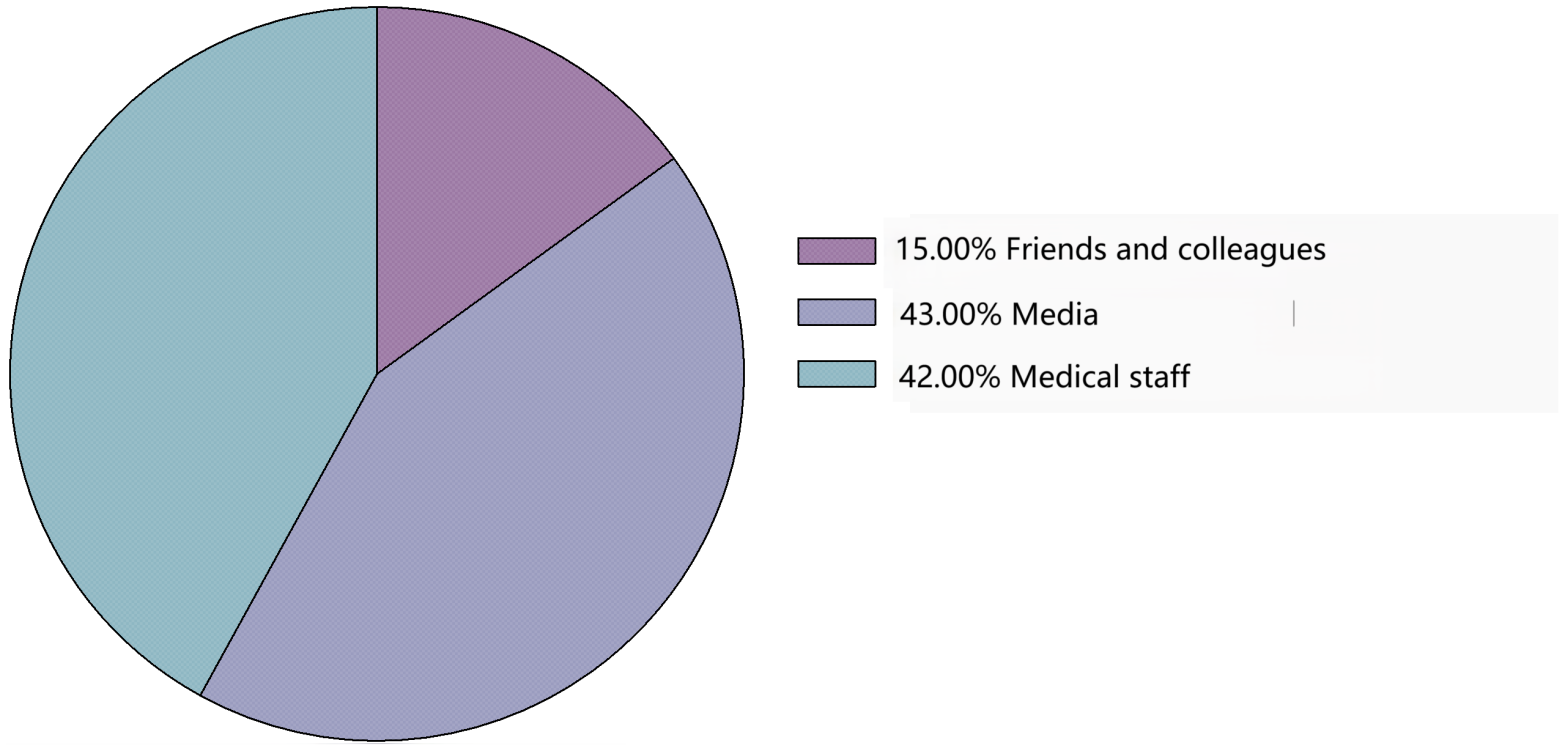
Sources of knowledge about lung cancer.

**Figure 2:**
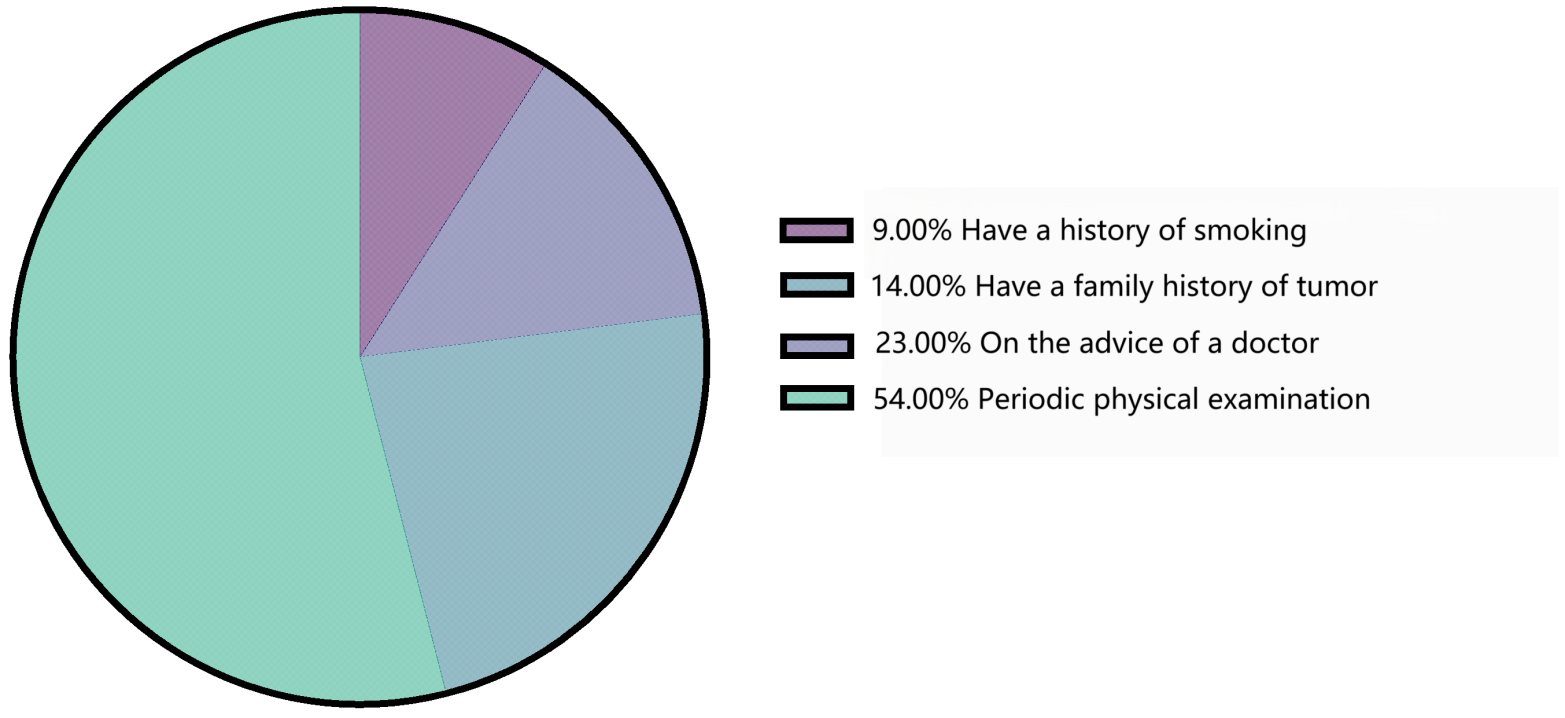
Reason for lung cancer screening.

A total of 31,184 respondents were included in this study. Based on their responses, only 7,446 (23.88%) participants had a good understanding of lung cancer (Table 1). The main sources of their knowledge about lung cancer were the media (43%) and medical staff (42%; Figure 1). Of the 31,184 respondents, 10,436 (33.5%) had undergone low-dose spiral CT (LDCT) screening for lung cancer (Table 1). Among these 10,436 respondents, the main reasons for undergoing LDCT screening were regular physical examinations (54%), followed by medical staff recommendations (23%; Figure 2).

**Table 1:** Basic situation of awareness about lung cancer.

Variable	Frequency (*n*)	Percentage (%)
Good understanding of lung cancer		
Yes	7,446	23.88
No	23,738	76.12
Lung cancer screening experience		
Yes	10,436	33.5
No	20,748	66.5
Total	31,184	100.0

### 3.2 Characteristics of the participants

Table 2 shows that the majority of variables exhibited significant differences between the two groups. The residents who were willing to choose county-level or lower-level medical institutions for lung cancer screening were more likely to have lower educational backgrounds, more stable careers, no commercial insurance, urban workers’ medical insurance, a lower monthly income, a negative family history of cancer, lower acceptable screening costs, and stable psychological status (*p*<0.05).

**Table 2:** Characteristics of the participants.

Variables	County-or lower-level hospitals (*n* = 7,198)	Higher-level hospitals (*n* = 23,986)	*χ*^2^/T	*p*-value
Age	40.45 ± 14.209	40.69 ± 13.640	−1.301	0.193
Sex [*n* (%)]			2.329	0.127
Male respondents	3,791 (23.4)	12,387 (76.6)		
Female respondents	3,407 (22.7)	11,599 (77.3)		
Ethnicity [*n* (%)]			3.665	0.056
Han	6,963 (23.0)	23,307 (77.0)		
Minority	235 (25.7)	679 (74.3)		
Education [*n* (%)]			408.130	<0.001
High school or lower	2,202 (30.9)	4,935 (69.1)		
College /Junior college	4,421 (21.9)	15,740 (78.1)		
Master’s degree or higher	575 (14.8)	3,311 (85.2)		
Occupation [*n* (%)]			132.733	<0.001
Government or public institution	2,752 (24.5)	8,460 (75.5)		
Private enterprises	3,481 (21.2)	12,702 (78.8)		
Self-employed entrepreneur	273 (19.7)	1,111 (80.3)		
Student or jobless	755 (30.6)	1713 (69.4)		
Medical insurance [*n* (%)]			6.164	0.013
Medical insurance for urban and rural residents	1944 (22.1)	6,838 (77.9)		
Basic medical insurance for urban workers	5,254 (23.5)	17,148 (76.5)		
Commercial insurance [*n* (%)]			97.808	<0.001
Yes	576 (16.4)	2,926 (83.6)		
No	6,622 (23.9)	21,060 (76.1)		
Monthly income [*n* (%)]			727.526	<0.001
<3,000 yuan	1,344 (34.5)	2,554 (65.5)		
3,000–4,000 yuan	1,650 (29.5)	3,938 (70.5)		
4,000–5,000 yuan	1,574 (24.7)	4,801 (75.3)		
≥5,000 yuan	2,630 (17.2)	12,693 (82.8)		
Acceptable screening costs [*n* (%)]			100.926	<0.001
<100 yuan	1,137 (32.3)	2,386 (67.7)		
101–200 yuan	1802 (28.1)	4,620 (71.9)		
201–300 yuan	2,250 (25.2)	6,671 (74.8)		
301–400 yuan	1,061 (20.9)	4,016 (79.1)		
>400 yuan above	948 (13.1)	6,293 (86.9)		
Smoking [*n* (%)]			0.135	0.713
Yes	1896 (23.2)	6,266 (76.8)		
No	5,302 (23.0)	17,720 (77.0)		
Family history of tumors [*n* (%)]			16.335	<0.001
Yes	1,142 (21.0)	4,300 (79.0)		
No	6,056 (23.5)	19,686 (76.5)		
Psychological status [*n* (%)]			19.583	<0.001
Stable	2,332 (24.8)	7,074 (75.2)		
Unstable	5,178 (23.8)	16,600 (76.2)		

### 3.3 Multivariate analysis

We included significant indicators from the univariate analysis in the multivariate analysis. The results of the binary logistic regression analysis are presented in Table 3. The participants with a strong understanding of lung cancer were more willing to undergo lung cancer screening at county-level or lower-level medical institutions (odds ratios = 1.182, 95%CI 1.110 to 1.259). The residents with unstable psychological status were less willing to choose these institutions (odds ratios = 0.849, 95%CI 0.800 to 0.902). In addition to a family history of lung cancer (*p* > 0.05), factors such as education, occupation, basic medical insurance, commercial insurance, monthly income, and acceptable screening costs were all significantly associated with the residents’ willingness to undergo lung cancer screening at county-level or lower-level medical institutions (*p* < 0.05).

**Table 3:** Factors associated with the choosing willing on lung cancer screening in situations.

**Variables**	**Experimental group**	**Comparative group**	**OR (95%CI)**	*P* **value**
Understanding of lung cancer	Good understanding	Bad understanding	1.182 (1.110,1.259)	<0.001
Psychological status	Unstable status	stable status	0.849 (0.800,0.902)	<0.001
Education	College school/Junior college	High school or lower	0.713 (0.669,0.761)	<0.001
	Master's degree or higher		0.523 (0.469,0.584)	<0.001
Occupation	Private enterprises	Government or public institution	0.812 (0.765,0.862)	<0.001
	Self-employed entrepreneurs		0.694 (0.600,0.802)	<0.001
	Student/jobless		1.036 (0.935,1.147)	0.502
Type of health insurance	Basic medical insurance for urban workers	Medical insurance for urban and rural residents	1.107 (1.041,1.178)	0.001
Commercial insurance	Yes	No	0.826 (0.749,0.911)	<0.001
Monthly income	3001~4000 yuan	<3000 yuan	0.884 (0.807,0.967)	0.007
	4001~5000 yuan		0.759 (0.693,0.832)	<0.001
	≥5000 yuan		0.578 (0.530,0.631)	<0.001
Family history of lung cancer	Yes	No	0.976 (0.907,1.051)	0.526
Acceptable screening costs	101~200 yuan	<100 yuan	0.826 (0.755,0.905)	<0.001
	201~300 yuan		0.726 (0.666,0.792)	<0.001
	301~400 yuan		0.592 (0.536,0.645)	<0.001
	>400 yuan		0.381 (0.345,0.422)	<0.001

### 3.4 Propensity score matching

We considered that binary regression could not infer causality as the regression results only represented the correlation. Since our study was cross-sectional, we used PSM to simulate the principles of a quasi-experiment to obtain results that closely aligned with causal inference. This approach allowed us to examine the effect of having knowledge of the disease on cancer screening decisions.

#### 3.4.1 Balance test in PSM

As mentioned above, psychological states can significantly influence decision-making behavior, a perspective confirmed by numerous studies (18, 19). During the propensity score matching process, we initially conducted a matching analysis on the overall population to validate the findings presented in Table 3. Subsequently, we divided the participants into groups based on their psychological states to assess the influence of lung cancer-related knowledge on the choice of medical institutions.

After using psychological status as a stratification variable, the two groups showed no significant differences in covariates following propensity score matching (*p* > 0.05). For the residents with unstable psychological status, the matched Pseudo-R^2^ value decreased from 0.008 to 0.002, the LR chi^2^ from 185.40 to 28.58, the mean bias from 5.6 to 2.0, and the median bias from 5.1 to 1.2, all indicating significant improvement. For the residents with stable psychological status, the matched Pseudo-R^2^ value decreased from 0.016 to 0.0021, the LR chi^2^ from 171.71 to 4.43, the mean bias from 8.7 to 1.4, and the median bias from 6.7 to 1.1. These results collectively suggest that the sample matching was successful (Table 4).

**Table 4:** Sample balance test after stratification by psychological status.

		Stable Psychological Status			Unstable Psychological Status		
Variable		Deviation Reduction (%)	T	*p*	Deviation Reduction (%)	T	*p*
Age	Unmatched	82.3	2.05	0.041	83.3	1.04	0.301
	Matched		0.29	0.77		0.15	0.884
Sex	Unmatched	58.7	0.2	0.84	84.7	−1.47	0.143
	Matched		−0.07	0.947		−0.19	0.852
Ethnicity	Unmatched	11.9	−0.24	0.812	87.4	−0.75	0.454
	Matched		−0.17	0.866		−0.08	0.936
Education	Unmatched	76.3	5.75	<0.001	88	7.57	<0.001
	Matched		1.13	0.26		0.77	0.443
Occupation	Unmatched	89.4	−6.73	<0.001	99.5	−6.77	<0.001
	Matched		0.61	0.544		−0.03	0.975
Medical insurance	Unmatched	30.9	−1.93	0.054	67.9	−2.72	0.007
	Matched		−1.06	0.288		−0.71	0.478
Commercial insurance	Unmatched	60.3	0.53	0.594	69.5	1.12	0.262
	Matched		0.17	0.866		0.28	0.781
Monthly income	Unmatched	80.1	3.12	0.002	90.1	4.92	<0.001
	Matched		0.5	0.617		0.42	0.678
Acceptable screening costs	Unmatched	78.7	5.68	<0.001	88.7	5.77	<0.001
	Matched		0.99	0.324		0.55	0.584
Smoking	Unmatched	64.2	3.25	0.001	78.6	2.91	0.004
	Matched		0.92	0.359		0.51	0.611
Family history of tumors	Unmatched	45.3	8.94	<0.001	66.7	6.3	<0.001
	Matched		0.37	0.714		1.62	0.105

#### 3.4.2 Average treatment effect on the treated

Table 5 shows the average treatment effect on the treated (ATT) of lung cancer knowledge on the respondents’ willingness to undergo lung cancer screening at county-level or lower-level medical institutions. The willingness percentage increased by 2.8% in the treated group after using kernel matching, 3.1% after using 1:1 nearest neighbor matching, and 2.4% after using radius matching.

**Table 5:** Average treatment effect on the treated (ATT) of lung cancer knowledge on the choice of medical institutions for screening, stratified by psychological status.

	Matching method	ATT	S. E	*Z*
All study participants	Unmatched	0.013^*^	0.006	3.10
	Kernel matching	0.028^*^	0.006	4.94
	1:1 nearest neighbor matching	0.031^*^	0.008	3.73
	Radius matching with caliper size 0.001	0.024^*^	0.005	5.32
Stable psychological status	Unmatched	−0.008	0.009	−0.81
	Kernel matching	0.007	0.009	0.78
	1:1 nearest neighbor matching	0.014	0.014	0.98
	Radius matching with caliper size 0.001	0.008	0.009	0.91
Unstable psychological status	Unmatched	0.032^*^	0.007	4.56
	Kernel matching	0.039^*^	0.007	5.62
	1:1nearest neighbor matching	0.053^*^	0.01	5.02
	Radius matching with caliper size 0.001	0.042^*^	0.007	5.98

In the kernel matching, the kernel function and broadband use default values. ATT, Average Treatment Effect on the Treated.

* Denotes statistically significant.

We then stratified the analysis by mental health status. Among the individuals with stable psychological status, the results were not statistically significant across the matching methods. In contrast, among those with unstable psychological status, the percentage of residents with good lung cancer knowledge willing to undergo screening at county-level medical institutions increased by 3.9% after using kernel matching, 5.3% after using 1:1 nearest neighbor matching, and 4.2% after using radius matching.

We used a bootstrap simulation with 500 replications to estimate the standard error of the propensity score for each unit.

## 4 Discussion

First, this study revealed that the residents’ knowledge about lung cancer in Sichuan Province was limited, with only 23.88% of the urban residents having a good understanding of the disease. In addition, 33.5% of the respondents reported having undergone lung cancer screening using LDCT. A multicenter, population-based, prospective cohort study in China conducted between 19 February 2013 and 31 October 2018 found that of the 1,016,740 participants enrolled, 79,581 high-risk participants underwent an LDCT scan, accounting for a proportion of only 7.8% (10). This proportion is significantly lower than that observed in the present study. We found that Sichuan Province has been vigorously publicizing the benefits of LDCT screening through many medical institutions and media channels since 2016. In addition, several lung cancer community screening programs have been organized by West China Hospital since 2019. This shows that Sichuan Province may be at the forefront in China in promoting LDCT screening for lung cancer among high-risk populations. Furthermore, our patients’ characteristics may also have caused the large difference between our results and those of previous studies. According to the findings of the National Lung Screening Trial, screening for lung cancer with LDCT is the most effective method for reducing lung cancer mortality (23). Therefore, there is an urgent need to improve patients’ knowledge about lung cancer, particularly by medical staff, as this may help to increase the lung cancer screening rate in high-risk populations.

Second, the multivariate and PSM analyses indicated that the residents with a strong understanding of lung cancer were more likely to choose county-level or lower-level medical institutions for screening. Their understanding of the disease reflects their health literacy. As one of the social determinants of health, health literacy represents the cognitive and social skills that motivate individuals to gain access to, understand, and use information to promote and maintain good health (24). Therefore, health literacy is essential for individuals to make informed healthcare decisions and benefit from healthcare services. Inadequate health knowledge is associated with high healthcare expenditures and the irrational use of healthcare services, as proven by several studies (25–27).

Finally, the stratified PSM analysis results suggested that psychological status may also influence residents’ choice of medical institutions. We found that the impact of lung cancer knowledge was more pronounced among those with unstable psychological status compared to the participants with stable psychological status. Previous research indicates that individuals with anxiety disorders exhibit significantly greater risk aversion (19). In our study, this result may be explained by the tendency of the anxious residents to choose institutions with lower rates of misdiagnosis or missed diagnoses in lung cancer screening to alleviate anxiety related to uncertain examination outcomes. This explanation aligns with the ATF theory introduced in the background (18, 19). The more substantial effect may also suggest that anxious residents have greater potential for improvement and that psychological status may play a positive mediating role between lung cancer knowledge and the choice of county-level hospitals. However, this hypothesis requires further validation through more detailed follow-up research. The findings suggest that measures to alleviate patient anxiety may play a role in enhancing the effectiveness of the hierarchical medical system and promoting rational patient flow.

This study has several potential limitations. It relied on self-reports to measure the residents’ knowledge of lung cancer, which may have introduced measurement errors. In addition, as the survey sites were mainly hospital-based physical examination centers, there may have been a population selection bias. Future studies should incorporate additional research design elements to address these limitations.

## Data Availability

The original contributions presented in the study are included in the article/Supplementary material, further inquiries can be directed to the corresponding authors.
